# Correlates of psychological functioning of homeless youth in Accra, Ghana: a cross-sectional study

**DOI:** 10.1186/1752-4458-9-1

**Published:** 2015-01-03

**Authors:** Kwaku Oppong Asante, Anna Meyer-Weitz, Inge Petersen

**Affiliations:** Discipline of Psychology, School of Applied Human Sciences, University of KwaZulu-Natal, Howard College Campus, Durban, 4041 South Africa; Department of Psychology, Regent University College of Science & Technology, Accra, Ghana

**Keywords:** Homeless youth, Mental health, Psychological functioning, Resilience, Substance use, Violent behaviours

## Abstract

**Background:**

Research on homeless youth has shown that this population is at high risk for various mental health problems. Previous studies conducted among homeless young adults in Ghana have focused primarily on economic, social and cultural causes of homelessness, their engagement in risky sexual behaviours and the prevalence of STI including HIV/AIDS. We are therefore not fully informed of the prevalence of psychological symptoms and their associated factors. The aim of the study was to determine the association between psychological functioning and social and health risk behaviours among a sample of homeless youth in Ghana.

**Methods:**

A cross-sectional survey of a convenience sample of 227 (122 male and 105 female) homeless youth was conducted in Ghana in 2013. An interviewer-administered questionnaire was used to collect data due to low level of literacy among the study population. Pearson-moment correlation coefficient (*r*) and multiple standard regression models were fitted to analyse the data.

**Results:**

Approximately 87% of the participants in this study exhibited moderate to severe psychosocial symptoms. Specifically, emotional, conduct, hyperactivity and peer relationship problems among the participants were 69%, 74%, 54% and 89% respectively. Overall psychosocial functioning was predicted by stigma (self-stigma and experienced stigma), violent behaviours and suicidal ideation. Substance use and perceived resilience were significantly associated with emotional problems.

**Conclusion:**

There is a need for holistic interventions to help improve the psychological and social functioning of homeless youth. Such programmes should strengthen socio-emotional coping strategies in street youth as well as address contextual risk factors such as stigma and discrimination by the public.

## Introduction

Poor mental health is a major cause of morbidity in low to middle income countries, with depression accounting for a large proportion of the disease burden [1]. Among the general population, several studies have shown that there are positive relationships between poor mental health and substance use, traumatic experience and sexual risk behaviours (unprotected sex and multiple sexual partners) [2–5].

There are significant developmental changes that take place during the transition from childhood to adolescence, which are accompanied by physical and psychological challenges [6]. Compared to other adolescents, these changes are more severe for street children who have to make this transition in the absence of financial, social and psychological support in their lives. It is thus not surprising that homeless youth have been found in high income countries to be at greater risk for mental health problems and engaging in high risk behaviours than those found in housed populations [7–9]. According to UNICEF [10], the rate of mental illness among homeless youth is very high, and being two times greater than youth in the general population [11, 12].

Various studies suggest that psychological well-being of homeless youth is associated with a variety of risk and protective factors. Risk factors are those characteristics of individuals that increase the likelihood of developing a mental health problem or increasing the severity of the problem, whilst protective factors on the other hand serve to modify or ameliorate the effects of mental health problems [13]. Among homeless youth population, risk factors to their mental health include number of years spent on the street [14, 15]; substance use [16, 17]; suicidal ideation [18]; stigma [19, 20] and physical and sexual abuse [11]. Perceived resilience [18, 19, 21] and social support [22–24] were identified as protective factors against various mental health problems. In their study of homeless youth in Canada, Cleverley and Kidd [18] revealed that perceived resilience was associated with less suicidal ideation and other life threatening behaviours. Similarly studies have suggested that perceived resilience among young adolescents may serve as a protective factor against health risk behaviour such as smoking, alcohol use and physically inactivity [21, 25]. There are currently no data regarding the psychological functioning and its associated factors within the African context. Most of the previous studies have been conducted from developed and economically resourced countries, particularly Canada and the United States.

The population of homeless youth is growing in cities such as Accra. Headcounts of street children ranged from 35,000 in 2009 [26] to 90,000 in 2013 [27]. Previous studies conducted among homeless young adults in Ghana have focused primarily on economic, social and cultural causes of homelessness [28, 29], their engagement in risky sexual behaviours and the prevalence of Sexually Transmitted Infections (STI) including HIV/AIDS [30–32]. While Wutoh et al. [32] found that homeless children were sexually active, and suffered from both physical and sexual abuse on the street, especially girls. However, the authors did not examine the psychological functioning of the participants in their study, although they did suggest the need for interventions that would address both mental health and risky sexual behaviour. In the context of this gap in knowledge on the psychosocial functioning of street youth in Ghana, the aims of this study were to determine i) the prevalence of behavioural and emotional problems among homeless youth in Accra; and ii) the factors associated with their psychological functioning. A better understanding of the psychological functioning of homeless youth and their correlates may help design appropriate interventions to improve the mental health status of this vulnerable and disadvantaged population.

### Theoretical framework

The risk and protective factors model [33] served as the theoretical framework to guide this study in relation to psychological functioning among vulnerable populations. This model outlines factors within a particular population that may ameliorate the effects of psychological problems (protective factors) or exacerbates the probability of developing a psychological problem (risk factors). Among vulnerable populations, several factors may be associated with susceptibility to mental health problems. These include personal and situational characteristics associated with mental health, as well as physical health, social resources and social support.

Among street connected children and youth, pertinent socio-demographic factors leading to compromised psychological functioning may include their younger age, years of living on the street, and lack of education. Poor health seeking behaviour, vulnerability to physical and sexual abuse and maladaptive coping strategies, including the use of drugs and alcohol, can have a huge long term effect on the mental health of homeless youth. Knowledge of risk and protective factors associated with psychological functioning of street children and youth is needed to help develop appropriate harm reduction programmes to this population.

## Methods

### Study site and participants

Participants comprised homeless children and adolescents in the Central Business District of Accra, the capital city of Ghana. This study area was selected because within Ghana, it has the second largest number of street children [26], with street children frequently found in places such as markets, bus and train stations [34]. A convenience non-probability sampling strategy was used to recruit homeless youth in 2013. For this cross-sectional survey, the sample consisted of 227 homeless children and adolescents, with ages ranging from 8-19 years with a mean age of 12.58 (SD = 2.51).

### Measures

A cross-sectional study was conducted which included the administration of the following measures:

**The Strength and Difficulties Questionnaire (SDQ)** [35], is an internationally validated screening tool for child and adolescent emotional and behavioural difficulties. It was used to assess the psychological functioning of the participants. The SDQ includes subscales for prosocial behaviour, hyperactivity/attentional, emotional, conduct and peer-relationship problems. The SDQ is rated on a 3-point Likert scale (Not True, Somewhat True, and Certainly True) with a score range of 0-40. The sum of the first four subscales gives the total psychological difficulties per child. Scoring is classified from 0 to 15 as normal (no psychological symptoms), 16 to 19 as borderline (moderate psychological symptom) and 20 to 40 as abnormal (severe psychological symptoms). Higher scores on the total SDQ scale reflect poorer psychological functioning. Acceptable reliability coefficients have been reported in Ghanaian sample [36, 37]. The Cronbach’s alpha for the SDQ was 0.72 in this study.

**Multidimensional Scale Perceived Social Support (MSPSS)** [38] was used to measure perceived social support along three dimensions: from the family, friends and significant others in the form of a 12-item, self-administered questionnaire. The scale is rated on a 5-point Likert type ranging from 5 (strongly agree) to 1 (strongly disagree). The MSPSS has been found to be reliable in various different samples internationally including Ghana [39]. Acceptable reliability coefficients of 0.89 have been reported in a Ghanaian sample [39]. The overall Cronbach’s alpha for the present study was 0.87.

**The Connor-Davidson Resilience Scale (CD-RISC)** [40], a brief self-rated assessment was used to help quantify resilience in a form of 25 items with responses in a form of 5-point Likert scale ranging from 0 (not true at all) to 4 (true nearly all the time). The tool includes five dimensions, which are focused on personal competence, tolerance of negative effects, adaptability, control and spiritual influences. The Cronbach’s alpha for the original scale was 0.89. The CD-RISC has been found to have good internal reliability with Cronbach’s alpha values ranging from 0.89 to 0.93. The total score on the CD-RISC ranges from 0-100, with higher scores reflecting greater resilience. A Cronbach’s alpha of 0.90 for CD-RISC was calculated in this study.

### Social stigma

This 12-item questionnaire developed by Kidd [20] was used to assess social stigma experienced by homeless street youth. The stigma scale includes items assessing the experience of being stigmatized, self-blame, and perception of public attitudes. These items are categorised into two main dimensions: self-blame (self-stigma) and experienced stigma. The social stigma scale has an overall internal reliability of 0.87, with the subscales of self-blame and experienced stigma having reliability coefficients of 0.78 and 0.89 respectively. The overall Cronbach’s alpha for the study was 0.90.

### Suicidal ideation

Four questions adapted from the South African Youth Risk Behaviour Survey [41] were used to assess the frequency of suicide-related thought over the past one month. A high score on this scale indicates higher levels of suicidal ideation. The Cronbach’s alpha for this scale in this study was 0.75.

### Substance abuse

Five questions adapted from the South African Youth Risk Behaviour Survey [41] were asked to assess substance use or abuse. These questions were posed to participants to elicit information about their engagement in substance use or abuse. The total score was computed by summing up the different items. Responses were coded such that higher score would indicate greater engagement in substance abuse. The Cronbach’s alpha for this scale in this study was 0.81.

### Violence-related behaviours

A violence scale was calculated from 11 questions which assessed various violent and violence-related behaviours among street children. These questions were adapted from the South African Youth Risk Behaviour Survey [41] and framed to measure specific behaviours related to violence, violence related and aggressive behaviours. A total score was created by summing up all 11 items from the violence measure. The dichotomous responses (Yes and No) scale were coded in such a way that a higher score represented higher levels of engagement in violence and violence related behaviours. The violence measure yielded an acceptable Cronbach’s alpha reliability coefficient of 0.72.

### Data collection and procedures

Two research assistants who were fluent and knowledgeable of the language spoken by the street youth were recruited and trained. The research participants were approached at specific designated places and asked whether they would be willing to participate in the study. The data was collected through an interviewer-administered questionnaire (as a result of low level of education) and most of the participants listened attentively as they were asked questions from the research instrument. It took an average of 30 minutes to administer the full questionnaire and data collection lasted for 8 weeks. The majority of the participants were interviewed in Twi and Ga (two predominant local languages spoken in Accra, Ghana). Each participant was compensated with a voucher worth approximately US$2.00 as a reward for participation in the study. While this amount may seem insignificant, this would enable the youth to buy a daily meal. None of the participants expressed the need for psychological service although they were told of the availability of a psychologist should they require such a service.

### Ethical consideration

Permission to conduct the study was granted from the Department of Social Welfare, Accra, Ghana and the University of KwaZulu-Natal Human and Social Science Ethics Committee (Ethical Approval number: HSS/1144/012D).

### Data analyses

The questionnaires administered to the participants were checked for completeness and data was entered into Microsoft Excel 2007 spreadsheet and imported into the Statistical Package for the Social Sciences version 21.0 for Window (IBM SPSS) for data analysis. Data quality was ensured by examining assumption of normality and homogeneity of variances. To determine the best predictors of psychological functioning, two analyses were conducted. First, the Pearson-moment correlation coefficient (*r*) was conducted to examine the relationship between psychological functioning, perceived resilience, suicidal ideation, violence behaviours, substance abuse, self-stigma, general stigma. The composite scores derived from the individual items measuring the various health risk behaviours was used in this analysis. Secondly, five standard regression fitted models were run using the total difficulty and its 4 domains (i.e. emotional problems, conduct problems, hyperactivity and peer problems). This was used to determine the best predictors of psychological functioning and its domains, and to ascertain the variables which made significant contribution in the regression models. Only predictors that had significant correlation coefficients with the criterion variables entered into the regression models.

## Results

### Demographics characteristics of the sample

The demographic characteristics of the sample are presented in Table 1. Males comprised approximately 54% of the sample, and approximately 80% were between the ages of 8-14. Over 59% of the participants indicated that poverty was the main reason for being homeless and about 26% had been abused (both physically and sexually). Over half (59%) of the participants had up to basic education level, and about 43% had lived on the street for 3-8 years. Significantly more females (19.8%) reported sexual abuse as the cause of leaving home than males (2.5%), *χ*^2^ (1, 223) = 22.87, p < 0.001.

**Table 1:** **Demographic characteristics of the sample**

Characteristics	***N***	%
**Gender**		
**Male**	122	53.7
**Female**	105	46.3
**Ages (M = 12.58, SD = 2.51).**		
**8 – 10 years**	50	22.4
**11-14 years**	129	57.9
**15 years and over**	44	19.7
**Previous level of education**		
**No formal education**	69	30.5
**Primary school (Grade 1–6)**	133	58.9
**Junior secondary school (Grade 7–9)**	24	10.6
**Years living on the street**		
**<1 year**	26	11.6
**1-2 years**	101	45.1
**3-5 years**	69	30.8
**5 years and more**	28	12.5
**Reasons for being homeless**		
**Family poverty**	132	59.2
**Dysfunctional problems**	15	6.7
**Maltreatment: Sexually abused**	23	10.3
**Maltreatment: Physical abused**	34	15.3
**Divorce**	12	5.4
**Other reasons**	7	3.1

N = Number, % = Percentage of N, M = Mean, SD = Standard Deviation.

### Psychological functioning of homeless youth

The general psychological functioning of the participants in the study is presented in Table 2. The overall difficulty score was very high (*M* = 22.0, *SD* = 6.05). Only 12.5% of the participants were not exhibiting any psychological symptoms, with approximately 87% exhibiting moderate to severe psychological symptoms. The results further revealed that of the sample, emotional problems was reported by 68.9% (*M* = 6.75, *SD* = 2.32); conduct problems by 73.8% (*M* = 5.04, *SD* = 2.87), hyperactivity/inattention problems by 53.9% (*M* = 5.39, *SD* = 2.14) and 88.6% reported peer relationship problems (*M* = 4.88, *SD* = 1.14) among the homeless youth.

**Table 2:** **Summary statistics for the SDQ (n = 227)**

				***Percentage in each SDQ category***		
Category	Mean	SD	Range	Normal	Borderline	Abnormal
**Total difficulty**	22.00	6.05	9-32	12.5	21.0	66.5
**Emotional symptoms**	6.75	2.32	0-10	32.0	16.6	53.4
**Conduct problems**	5.04	2.87	0-10	26.2	12.0	61.8
**Hyperactivity/inattention**	5.39	2.14	0-9	46.1	21.6	32.3
**Peer relationships problems**	4.88	1.41	2-10	11.4	54.1	34.5
*Prosocial behaviour	5.42	1.82	0-10	43.6	32.1	24.3

*This sub-scale is excluded from the computation of total difficulty score per participant.

### Relationship between psychological functioning and other study variables

The Pearson-moment correlation coefficient (*r*) was conducted to examine the relationship between psychological functioning, perceived resilience, suicidal ideation, violence behaviours, substance abuse, self-stigma, and general stigma. Table 3 shows small to moderate correlation coefficients for the predictor variables that are associated with total difficulty and its 4 domains. A significant positive relationship existed between overall total difficulty of a participant and suicidal ideation *(r* = 0.24; *p* < 0.001), violent behaviour (*r* = 0.14; *p* < 0.05), self-stigma (*r* = 0.33; *p* < 0.001) and experienced stigma (*r* = 0.15; *p* < 0.05). These results suggest that higher scores on the total SDQ scale (reflecting poor psychological functioning) were associated with increased levels of violence behaviour, self-stigma and experienced stigma.

**Table 3:** **Correlation matrix between psychological functioning and other study variables**

	Variables	1	2	3	4	5	6	7	8	9	10	11
1	**Total difficulty**	1										
2	**Emotional problems**	.75***	1									
3	**Conduct problems**	.73***	.29***	1								
4	**Hyperactivity**	.79***	.53***	.39***	1							
5	**Peer problems**	.30***	.09	.08	.09	1						
6	**Resilience**	.13	-.36***	.06	-.08	.04	1					
7	**Suicide ideations**	.24**	.30***	.16*	-.03	.03	-.55***	1				
8	**Substance abuse**	.10	.23**	.07	.07	.12	-.43***	.45***	1			
9	**Violent behaviour**	.15*	-.14	.36***	.05	.19**	-.13*	.18*	.51***	1		
10	**Self-stigma**	.33***	.47***	-.02	.38***	.06	.09	-.24***	-.01	-.31***	1	
11	**Experienced stigma**	.15*	.40***	-.13	.15*	-.02	.09	.18**	.03	.29***	.80***	1
12	**Social support**	.14	-.05	.47***	-.01	.17*	.12	-.19**	-.26**	.09	-.18*	-.22**

*p < .05; **p < .01; ***p < .001.

The following analyses were also done on the sub-scale of the SDQ and their relationship with the various independent variables. Emotional problems in homeless youth correlated positively with suicidal ideation (*r* = 0.30; *p* < 0.001), substance abuse (*r* = 0.23; *p* < 0.01), self-stigma (*r* = 0.47; *p* < 0.01) and general stigma (*r* = 0.40; *p* < 0.01). A negative correlation was found between emotional problems and perceived resilience (*r* = − 0.36; *p* < 0.001). The results imply that as emotional problems increase, homeless youth are more likely to report higher levels of suicidal ideation, general and self-stigma and elevated substance use. The results further suggest that higher levels of emotional problems were associated with lower levels perceived resilience.

Conduct problems positively correlated with suicidal ideation (*r* = 0.16; *p* < 0.01), violent behaviour (*r* = 0.36; *p* < 0.01) and social support (*r* = 0.47; *p* < 0.01). The results suggest that high levels of conduct problems were associated with high levels of suicidal ideation, violent behaviour and social support. There was a positive relationship between hyperactivity and self-stigma (*r* = 0.38; *p* < 0.01). Similarly, hyperactivity correlated with general stigma (*r* = 0.15; *p* < 0.05). This implied that as hyperactivity levels increase, both self-stigma and experienced stigma increases. The results as presented in Table 2 show that peer/relationship problems were positively associated with violence behaviour (*r* = 0.19; *p* < 0.01) and negatively related to social support (*r* = − 0.17; *p* < 0.05).

### Predictors of psychological functioning

To determine the predictors of total difficulty (total score on the SDQ) and its domain, five (5) regression models were conducted, using only predictors/independent variables that had significant relationship with the criterion variable in Table 3. The first regression model used the overall score of SDQ (psychological functioning) as criterion, and the second to fifth models included the domains of SDQ as criteria. The predictors included in the regression analysis were perceived resilience, suicide ideation, substance abuse, violence behaviour, self-stigma, experienced stigma and social support. The results of the analysis are presented in Table 4.

**Table 4:** **Summary of multiple regression of the best predictors of psychological functioning and its domains**

Models/Criterion variables	Predictors	Collinearity statistics						
		B	***SE B***	***β***	***t***	***R*** ^***2***^	***F***	Tolerance
**1. Total difficulty**	**Experienced stigma**	2.02	.43	.55	4.67***			.346
	**Violent behaviour**	.57	.14	.29	4.01***	.220	12.74***	.887
	**Self-stigma**	.27	.14	.23	2.00*			.358
	**Suicide Ideations**	.99	.33	.22	2.99**			.886
**2. Emotional problems**	**Experienced stigma**	.61	.16	.42	3.77***			.390
	**Resilience**	-.03	.01	-.27	-3.46***			.791
	**Violent behaviour**	.20	.07	.26	3.12**	.391	16.91***	.688
	**Substance use**	.33	.13	.23	2.58*			.646
	**Self-stigma**	.06	.05	.13	1.16			.397
**3. Conduct problems**	**Violent behaviour**	.44	.08	.46	5.46***	.336	25.31***	.650
	**Social support**	.12	.03	.35	4.69***			.846
	**Suicide Ideations**	.39	.15	.22	2.58*			.618
**4. Hyperactivity**	**Experienced stigma**	.93	.13	.72	6.98***	.200	27.17***	.361
	**Self-stigma**	-.17	.04	-.43	-4.15***			.361
**5. Peer problems**	**Violent behaviour**	.01	.03	.21	2.99**	.065	7.31**	.993
	**Social support**	.03	.01	.19	2.62**			.991

*p < .05; **p < .01; ***p < .001.

In Model 1, the results showed a significant joint influence of self-stigma, experienced stigma, violent behaviour and suicidal ideation on overall psychological functioning, (*R*^2^ = 0.22, *F* = 12.74; *p* < .001). The results indicated that 22% of the variance in psychological functioning could be explained by the predictors. The second model showed a significant joint effect of experienced stigma, perceived resilience, substance use and violence behaviour on emotional symptoms, (*R*^2^ = 0.391, *F* = 16.91; *p* < .001), and explained 39.1% of the variance in emotional problems. The third model revealed a significant joint influence of three (3) predictors: suicidal ideation, violence behaviour and social support on conduct problems, (*R*^2^ = 0.336, *F* = 25.31; *p* < .001), and explained 33.6% of the variance in conduct problems. The fourth regression model indicated that both self-stigma and experienced stigma had a significant joint influence of the predictors of hyperactivity, (*R*^2^ = 0.20, *F* = 27.17; *p* < .001), and explained 20% of the variance in hyperactivity. The fifth model revealed that violent behaviour and social support were the significant predictors of peer problems, *R*^2^ = 0.065, *F* = 7.31; *p* < .01, explaining only 6.5% of the variance in peers problems.

## Discussion

This study was conducted to examine the prevalence of psychological problems, and to determine factors that predicted these psychological problems among homeless youth. The results showed that approximately 87% of the participants in this study exhibited moderate to severe psychological symptoms. Overall psychological functioning was predicted by stigma (self-stigma and experienced stigma), violent behaviour and suicidal ideation. Substance use and perceived resilience were significantly associated with emotional problems.

The prevalence of emotional, conduct, hyperactivity and peer relationship problems among the participants were 69%, 74%, 54% and 89% respectively. These findings suggest that homeless youth in this study experienced poor mental health. This is consistent with previous studies conducted in developed resourced countries [22, 42, 43]. Several studies from high-income countries show risk factors for psychological functioning of homeless youth. A literature search was unable to access any similar studies on the risk factors for poor psychological functioning of street youth within sub-Saharan Africa. The findings of this study showed that overall psychological well-being was influenced by experienced stigma, self-stigma, suicidal ideation and exposure to violence. This is suggestive of the fact that where these factors are present, the psychological functioning of a street youth might be compromised.

Experienced and self-stigma were found to be associated with the poorer psychological functioning of homeless youth in this study. These finding lend support to a previous study conducted in the United States of America which found that perceived discrimination and negative stereotypic behaviours towards street youth influenced their mental health contributing to higher levels of loneliness, social alienation and depression [19, 20]. According to Crocker, Major and Steele [44] individuals who are stigmatized often have a characteristic that is not valued by a particular society and stigma directly affected the mental health of homeless youth in this study. Research in Ghana has shown that public perception to street youth is very hostile and undesirable [45]. In the study of public perception about street youth among various stakeholders in Ghana, Quashie [45] revealed a bleak picture as street youth are perceived as drug users, and thieves who are involved in petty criminal activities. This is further compounded by use of derogative and belittling words such as “*kubolo*” (a derogatory and belittling word used for street child in the Greater Accra region of Ghana). Given the literature, it is possible to indicate that stigmatization (whether self-stigma or perceived stigma by society) has a deleterious effect on homeless youth and reflects the extent to which stigma and discrimination of street youth directly impact the mental health of homeless youth. Discrimination and negative stereotypic attitudes by the public towards street youth influence their mental health and contribute to higher levels of loneliness, social alienation and depression. This finding also suggest that putting in place an anti-stigma and discriminations campaign, would lead to improvement in the psychological health in this vulnerable population.

The findings of this study also indicate a significant positive independent influence of suicide ideation on total psychological difficulty, suggesting that higher levels of suicidal ideation correspond to poor psychological functioning. This finding supports the research of Frederick et al. [46] who found that homeless youth who had a diagnosis of a mental disorder were twice as likely to experience suicidal ideation and suicidal attempts than those without such diagnosis. Previous studies in Ghana have shown street youth to be adaptable in the face of adversity [47]. However, there may be limits to this adaptability, as the cumulative effect of abuse, substance use, public stigma and other health risk factors affecting their psychological functioning, may lead them to have suicidal thoughts.

The finding of this study showed that violent behaviour was associated with lower levels of psychological functioning. Violent behaviour measured included having abused, having been beaten or coerced (such as been forced to have sex with someone). These forms of maltreatment have also been reported as reasons why homeless youth left home in Ghana [31], and are re-enacted on the street, with boys more likely to suffer from physical assault and girls more likely to be sexually abused or raped [31, 42, 48]. Our findings indicate that those exposed to severe forms of abuse and violence displayed higher number of psychological symptoms. This finding corroborates previous studies that draw an association between violence behaviours of homeless youth and mental health problems [49, 50]. For example, using a purposively selected sample of 601 homeless youth in Denver, USA, [49] found physical and sexual assault to significantly predict mental health outcomes such as major depressive symptoms and PTSD. Similarly, a high rate of comorbid diagnosis of post-traumatic stress disorder (PTSD) has been found in homeless youth who have been assaulted or injured by a weapon on the street [50]. Our findings thus corroborate the literature to suggest that engagement in violent behaviours on the street may have mental health consequences for homeless youth.

Resilience is a complex construct that involves interaction between adversity and an individual’s internal and external protective factors and competencies that allow one to overcome adversity [51]. According to some researchers resilience is considered as positive outcomes despite the experience of adversity, continued positive or effective functioning in adverse circumstances [40, 52]. Thus resilience can be considered as the ability to ‘bounce back’ in the face of adversity [40, 52]. Perceived resilience as measured by the CD-RISC, revealed a negative relationship with emotional problems, suggesting that higher perceived resilience was associated with lower emotional problems. This finding corroborates previous studies that report resilience as being a protective factor against the onset of various mental health problems [18, 21, 25] and provides support for the protective model of resilience that suggests that protective factors assist in neutralizing the effect of risk, thus reducing the impact of a negative outcome [53]. This finding also suggest that participants in this study are resilient, and that programmes aimed at building resilience in youth to cope better with the stressors of living on the streets should engage youth as early as possible when they become homeless to decrease the degree of deterioration in physical and mental health.

Substance use increases the likelihood of individuals engaging in risky sexual behaviours, such as non-condom use and multiple sexual partners [54] and heightens the probability of having psychological problems. Substance use was found in this study to be positively related to higher emotional problems. This supports previous research that has shown that substance use, including alcohol and hard drugs, are associated with greater emotional distress [23, 55]. Substance use among the homeless population has, however, also been reported to be a coping strategy [16, 17].

Social support was found to be positively associated with both conduct problems and peer relationship problems among homeless youth. In the absence of support from mainstream society like family and relatives, homeless youth rely on peers and “street family” for support to cope with stressful events on the street. However, these support systems have been shown to further entrench them into street life thereby putting them at greater risk for mental health related problems [56]. While gangs provide social support they also influence members to engage in anti-social behavior, hence the positive relationship between social support and conduct problems. These findings corroborate past studies that have indicated that social support available to homeless youth on the street could have deleterious effect on mental health [56, 57]. This finding shows that although participants had adequate support on the street, these available supports can lead to compromised social functioning. There is therefore the need to develop health enhancing social networks that provide homeless youth with alternative networks for gaining social support rather than support from deviant youth groups who promote anti-social behavior.

The findings of this study must be interpreted with caution, as several issues might have introduced bias in this study. The cross-sectional nature of the research did not allow cause-and-effect relationships to be established. Longitudinal research, although challenging especially with transient populations such as homeless youth may be necessary to help determine the trend of the relationships between the variables identified in this study. Second, the practical significance of the relationships observed in this study might be limited as most of the correlation coefficients were small or moderate in strength. Third, we measured only psychological symptoms, and therefore could not determine the prevalence of specific mental disorders such as depression and Post-traumatic Stress Disorder (PTSD). The need for future studies to examine the prevalence of specific disorders among this population is highlighted. Fourth, administering the questionnaire using an interviewer may have resulted in under reporting of behaviours such as suicide ideation, violent behaviour and substance use. Finally, most of the measures used were modified from the South African Youth Risk Behaviour Survey [41]. During modification and translation, it is possible, that the validity and reliability of these measures may have compromised. Notwithstanding this, most of the measures yield acceptable Cronbach’s alpha reliability coefficients of 0.70 and above.

## Conclusion

This study highlights that the majority of street youth in Ghana display moderate to severe psychological symptoms. The need for mental health services to help youth cope with multiple mental health problems is thus highlighted. The findings revealed that risk factors for poor mental health amongst street youth include experienced stigma, self-stigma, suicidal ideation and violence behaviour. Using an ecological systemic framework [58], the need for multilevel prevention interventions is highlighted. Firstly, at the individual level, there is a need for programmes aimed at building resilience in youth to cope better with the stressors of living on the streets, for example, through access to psychological counselling to address mental health issues and to develop better coping strategies to deal with past and current adversities. Secondly, at the interpersonal level there is a need to develop health enhancing social networks that provide homeless youth with alternative networks for gaining social support rather than support from deviant youth groups who promote anti-social behavior. Lastly, at the community and societal levels there is a need for programmes to address the social determinants of their poor mental health. In this regard, violence and harm reduction programmes, including early parenting programmes to reduce exposure to violence in the home, as well as anti-stigma campaigns are needed.
